# A two-way interface between limited Systems Biology Markup Language and R

**DOI:** 10.1186/1471-2105-5-190

**Published:** 2004-12-07

**Authors:** Tomas Radivoyevitch

**Affiliations:** 1 Department of Epidemiology and Biostatistics, Case Western Reserve University, Cleveland, Ohio 44106 USA

## Abstract

**Background:**

Systems Biology Markup Language (SBML) is gaining broad usage as a standard for representing dynamical systems as data structures. The open source statistical programming environment R is widely used by biostatisticians involved in microarray analyses. An interface between SBML and R does not exist, though one might be useful to R users interested in SBML, and SBML users interested in R.

**Results:**

A model structure that parallels SBML to a limited degree is defined in R. An interface between this structure and SBML is provided through two function definitions: write.SBML() which maps this R model structure to SBML level 2, and read.SBML() which maps a limited range of SBML level 2 files back to R. A published model of purine metabolism is provided in this SBML-like format and used to test the interface. The model reproduces published time course responses before and after its mapping through SBML.

**Conclusions:**

List infrastructure preexisting in R makes it well-suited for manipulating SBML models. Further developments of this SBML-R interface seem to be warranted.

## Background

Systems biology markup language (SBML) is a standard for representing dynamical systems of biological interest [1,2]. Interfaces between SBML and high level computational environments are currently being developed for Mathematica [3] and Matlab [4], but to the author's knowledge, no such efforts are being carried forth for R/S-plus. This brief paper presents the author's initial developments toward a two-way SBML-R interface. The interface is currently limited in the range of SBML input files that it can handle. For example, it only handles SBML level 2 and does not handle "Events" and "FunctionDefinitions." The interface can nevertheless be used for some models, examples [5,6] of which are included under "demo" in the SBMLR package [7]. This paper provides an explicit example of one approach to an SBML-R interface. It is assumed throughout that the reader is already quite familiar with both SBML [8] and R [9].

## Implementation

The software exists completely in R. It is comprised of four functions and is currently being distributed as a developmental package called "SBMLR" through Bioconductor [10]. The software was written subject to two constraints: 1) models expressed in SBML-like R must be exchangeable with a range of SBML models; and 2) models must be amenable to simulation in R. The first subsection that follows defines an SBML-like R model structure, the second illustrates how it can be used in simulations, and the third describes its conversions into and out of SBML.

### An SBML-Like Model Structure in R

**Figure 1:**
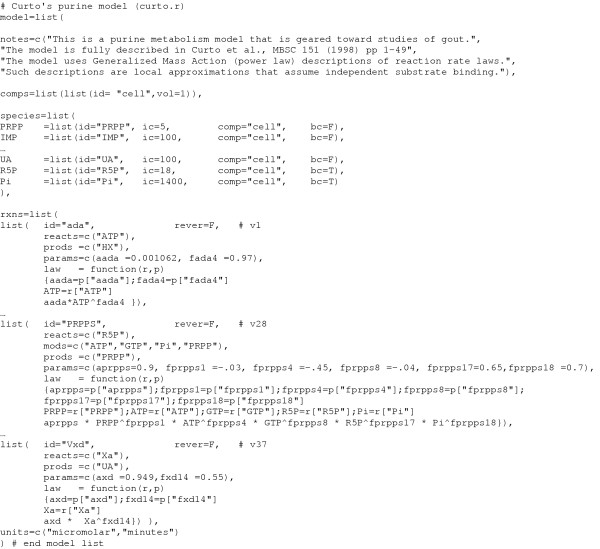
The model of Curto *et al. *implemented as an SBMLR structure

To facilitate mappings between SBML and R, an SBML-like list structure is defined in this subsection using the purine metabolism model of Curto et al. [6] as a specific example (Figure 1). In this figure and elsewhere, ellipses (...) indicate missing code not critical to current discussions; complete source codes are available through the SBMLR package [7]. The essential components of an SBML model, namely, its compartments, species and reactions, are all present in this R analog of an SBML model. In the model of Curto et al. [4], there is one compartment, be it the cell or the entire human body, and 18 species: 2 boundary conditions (bc = True) and 16 state variables (bc = False), each with an initial condition (ic) or value. Each reaction is a list that includes a reaction id, the names of species that are reactants (reacts), the names of species that are reaction rate modulators (mods), the names of species that are produced by the reaction (prods), parameter values (params), and the reaction rate law (law) function definition. In this framework, only state variables need be listed as products, boundary condition reactants can equivalently be listed as modulators, and missing terms (e.g. mods in reactions 1 and 37) are equivalent to a NULL assignment. The rate law function has as its input arguments two vectors, one carrying the concentrations of reactants and modulators (r), the other carrying reaction parameter values (p). If the body of the rate law function contains *n *statements, the first *n*-1 trivially convert input vector components into variables with the same names. The *n*th statement then contains the complete reaction rate law. It can occupy multiple lines, but it must be a single statement, i.e. it cannot depend on substitution variables temporarily defined in preceding statements.

### SBML-like Model Execution in R

**Figure 2:**
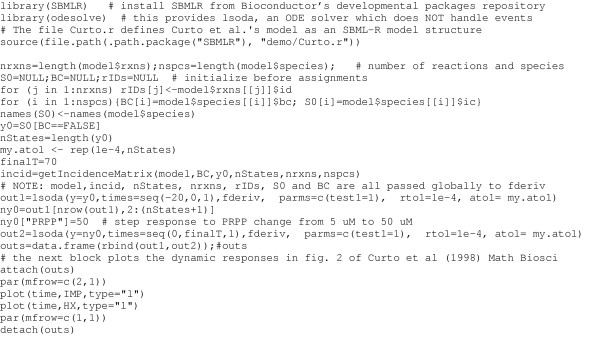
The purine metabolism model of Curto *et al. *represented in SBMLR Figure 1) simulated to respond to a 10-fold increase (5 μM to 50 μM) in phosphoribosylpyrophosphate (PRPP) at time *t *= 0

Model definition codes such as that given in Figure 1, when placed in a separate file (e.g. Curto.r), can be sourced into a parent script to become globally available for simulations. For example, the purine metabolism model of Curto et al. [6] can be simulated using the execution code shown in Figure 2.

**Figure 3:**
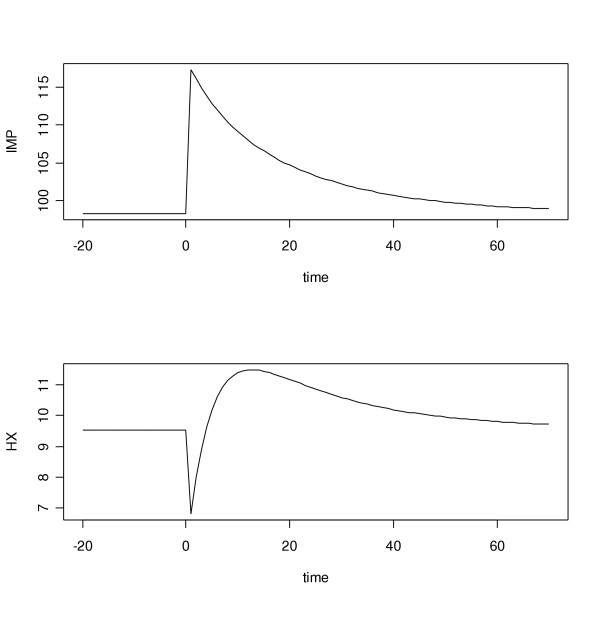
The purine metabolism model of Curto *et al. *responding to a 10-fold increase in phosphoribosylpyrophosphate (PRPP) at time *t *= 0 (see Figure 2). IMP is inosine monophosphate, HX is hypoxanthine, time is in minutes, and concentration is in μM.

**Figure 4:**
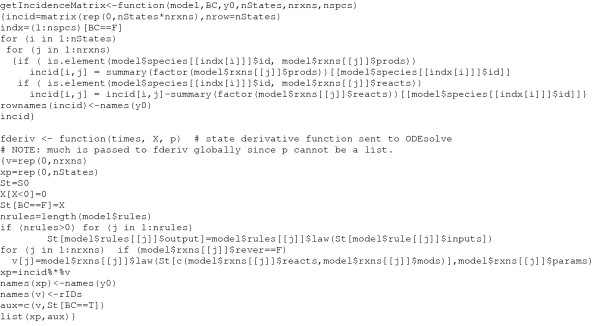
R codes for the functions getIncidenceMatrix() and fderiv().

This code simulates the response to a 10-fold increase in phosphoribosylpyrophosphate (PRPP) at time *t *= 0 and plots the responses of inosine monophosphate (IMP) and hypoxanthine (HX) as shown in Figure 3. Two functions called by this script are defined in the SBMLR package and shown in Figure 4. They are, getIncidenceMatrix(), which computes the incidence/stoichiometry matrix used by the second function, fderiv(), which computes state derivatives for integration by the function lsoda() of the "odesolve" package. In getIncidenceMatrix(), the incidence matrix is generated automatically using an *i *loop over the rows (i.e. state variables) and a *j *loop over the columns (i.e. reactions). If a state is a product of a reaction, the corresponding matrix element becomes a positive integer equal to its stoichiometry [factor() converts string names to factors so that summary() can count them], and similarly for reactants, though with negative numbers entering the matrix in this case (or possibly zero, if a reactant of a reaction happens to also be a product of the same reaction).

The function fderiv() creates the current species vector by overriding initial states with current states clipped to positive values, and by overriding any time varying boundary conditions defined by rules (SBML rules are not needed for the purine model, but are needed to implement other models [5]). The function fderiv() then computes the reaction rate flux vector (v) based on the current species vector (St) and multiplies it by the incidence matrix to produce the current state derivative vector (xp). The names of xp and v are reset at the end of each function call to override the problem of variables gaining new composite names from the names of their expression arguments.

### A Two-Way Interface between SBML and R

**Figure 5:**
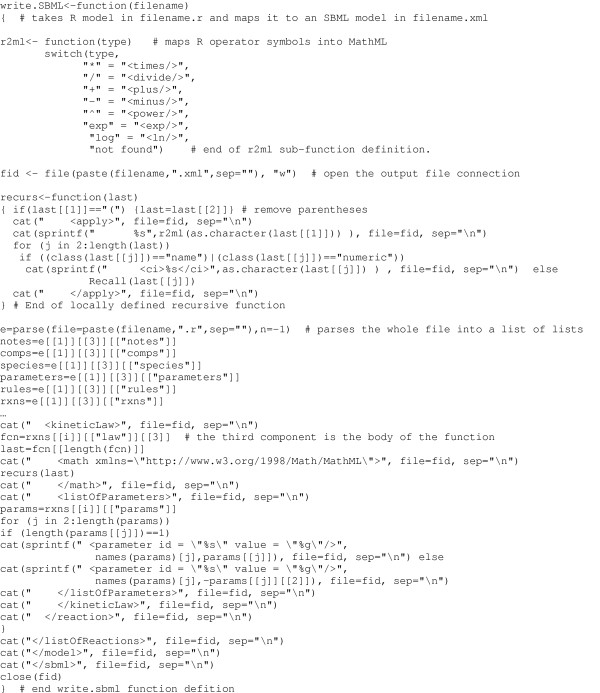
R code for the function write.SBML().

Two functions comprise the SBML-R interface: write.SBML() converts SBML-like R models (e.g. Curto.r) into SBML models (e.g. Curto.xml), and read.SBML() converts SBML models (e.g. Curto.xml) into an SBML-like R model (e.g. CurtoX.r). A key component of these two interface functions is a locally defined recursive function named recurs(). This function converts arbitrary R expressions into arbitrary MathML expressions, and vice-versa; it is defined differently, locally, in each of the two functions. In write.SBML(), shown in Figure 5, recurs() initially takes as its input argument the last component of the body of the kinetic rate law function definition, which is the entire rate law expression (as mentioned above, rate laws involving multiple R statements are not supported). In R, expressions are LISP like in that they contain a first element, the operator, and the remaining elements, the arguments, any of which can be an expression. If the operator is the parentheses operator, the action taken is that of a unary identity operator, and we simply skip it and move on to its argument since parentheses are not needed in MathML. Each nested call to the function recurs() sends "<apply>" and the converted operator to the output file on its way in, and a matching "</apply>" on its way out. Nested calling continues until all nodes of the expression tree are of class "name" or "numeric," i.e. when all found objects are leaves of the tree rather than "expressions" that require further parsing. Leaves are then sent to the output file bracketed by <ci> and </ci>.

**Figure 6:**
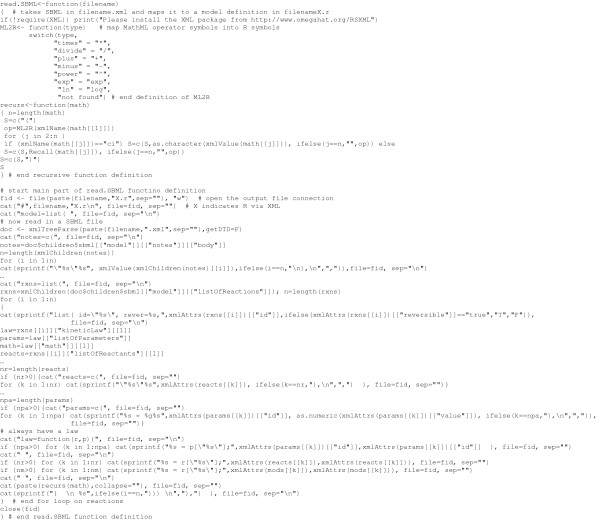
R code for the function read.SBML().

The second of the two SBML-R interface functions, read.SBML(), maps a limited range of SBML level 2 files (function definitions and events are not handled) into SBML-like R model files. Portions of read.SBML() are given in Figure 6. The main difference between this function, read.SBML(), and the previous function, write.SBML(), is that here, rather than using parse() to decompose the list-of-lists structure of the model defined in R, the SBML model is instead decomposed as an XML object using xmlTreeParse() of the XML package available to R [11]. In read.SBML(), the locally defined recursive function recurs() uses an overkill of parentheses to avoid operator precedence issues. This recursive function is passed a MathML reaction rate law which it parses recursively until the leaves of the tree (the "ci") are all found. During the recursion a corresponding R expression is built as a vector of character strings which, upon exit from the last of the recursive calls, is collapsed into a single string and sent to the output file as the last line of the current rate law function definition.

## Results

The function write.SBML() was applied to Curto.r to generate Curto.xml and the function read.SBML() was then applied to Curto.xml to generate CurtoX.r. Execution of the script given in Figure 2 with line 4 of the execution code changed to act on CurtoX.r instead of Curto.r generated the same plots as before (Figure 3). This shows that the R model was successfully converted into an SBML file that can be reconverted back into a properly functioning R model. The intermediate file Curto.xml was successfully validated as an SBML level 2 file [12]. The SBML file could thus be imported into visualization packages such as JDesigner [13].

## Discussion

**Figure 7:**
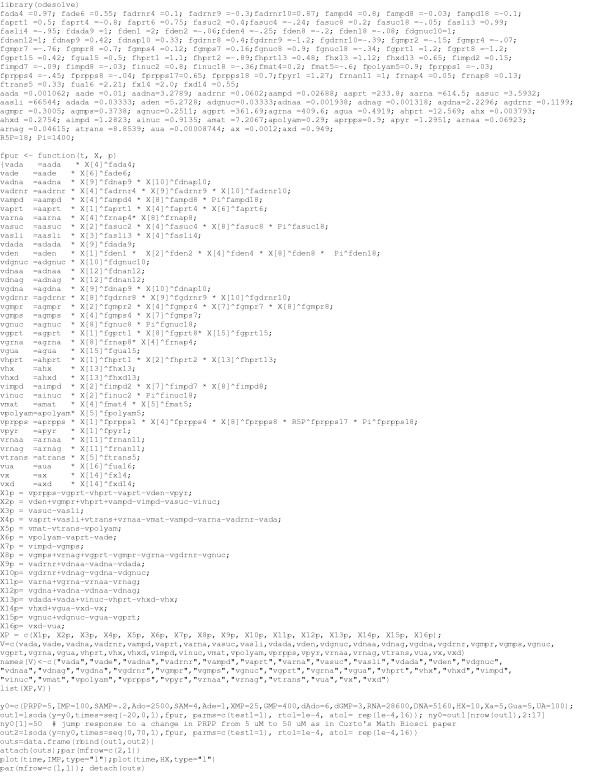
The purine metabolism model of Curto *et al. *implemented in "natural R".

If the model of Curto et al. [4] were implemented in R without any knowledge of SBML, a form that it might take is that given in Appendix B (Figure 7). Compared to its SBML-like counterparts, this code is more compact and easier to understand, e.g. the system's network connectivity is clearly visible. The disadvantage of such code is that it is not readily converted into SBML. Since the benefits of SBML are compelling, this disadvantage alone warrants the use of SBML-like model structures.

As SBML evolves to handle a broader range of dynamical systems, it will become more and more challenging for simulation packages to handle all possible SBML models. It is envisioned here that the development of this SBML-R interface will be driven by its users, and not by the model representation capabilities of SBML, i.e. it is expected that the users of this interface will be programmers who are capable of modifying it as their needs require.

## Conclusions

Compared to Matlab, which may be better equipped than R to simulate arbitrarily complex dynamical systems, R has the advantage of list handling infrastructure in parse() and xmlTreeParse(), and it also has the advantage of indexing by names instead of numbers. A further advantage, though not exploited here, is that R is object-oriented; in future versions of this interface, a print() method might be defined for objects of class SBMLR (i.e. models) to generate more readable renderings of models in R. Another advantage of R over Matlab is that it provides access to a much broader collection of microarray analysis tools, e.g. see Bioconductor [10]. This aspect is important for those individuals who are interested in biochemical systems analyses of microarray data [14,15]. For statisticians already familiar with R, there are also the obvious economies of maintaining system familiarity. Finally, perhaps the biggest advantage of R over Matlab is that it is freely available. On balance, there seems to be ample motivation for further developments of this interface between SBML and R.

## Availability and requirements

Project name: SBMLR

Project home page: 

Operating system(s): Windows XP

Programming language: R 2.0

Other requirements: R packages: XML and ODESOLVE

License: GNU GPL

Any restrictions to use by non-academics: no restrictions

## List of abbreviations

SBML = Systems Biology Markup Language; XML = extensible markup language; MathML = Mathematical Markup Language; ODE = ordinary differential equation.

## Authors' contributions

TR is the sole contributor.

## Appendix A

The SBMLR package is available through Bioconductor as a developmental package [7]. It has been developed and tested only under Windows XP. To install, do NOT unzip the file SBMLR.zip after downloading to a local directory, rather, within the R GUI, click *packages *and *install from local zip*. The XML package installs similarly [11]. Note that an error message from library(XML) can be resolved by copying the *.dll files of the XML package libs directory into the "C:\windows" directory. The ODESOLVE package must be installed before running simulations. This package is installed from the R GUI by clicking *packages *and *install from CRAN*.

## Appendix B

The implementation of Curto et al.'s model shown in Figure 7 is independent of any knowledge of SBML. It is included here to illustrate what comes "naturally" when implementing a model in R, see Discussion.
